# Bionic effects of nano hydroxyapatite dentifrice on demineralised surface of enamel post orthodontic debonding: in-vivo split mouth study

**DOI:** 10.1186/s40510-021-00381-5

**Published:** 2021-11-01

**Authors:** Purva Verma, Srirengalakshmi Muthuswamy Pandian

**Affiliations:** Department of Orthodontics and Dentofacial Orthopedics, Saveetha Dental College and Hospitals, Saveetha Institute of Medical and Technical Sciences, Saveetha University, 162, Poonamallee High Road, Velappanchavadi, Chennai, Tamil Nadu 600077 India

**Keywords:** Nano hydroxyapatite, Dentifrice, Surface roughness, Debonding, Atomic force microscope

## Abstract

**Background:**

Orthodontic debonding procedure produces inevitable enamel surface alterations, sequelae to which are enamel demineralization, sensitivity and retention of pigments. Several agents have been employed to counterbalance the same. The purpose of this study was (1) To evaluate the hypothesis that no significant difference exists in the remineralising potential of nano hydroxyapatite (NanoHAP) dentifrice and fluoridated dentifrice after orthodontic debonding, (2) To estimate the enamel topographic parameters following use of nano HAP dentifrice, post orthodontic debonding.

**Methods:**

Sixty upper first bi-cuspids (30 subjects) planned for therapeutic extraction for the orthodontic treatment were bonded with a light cured adhesive. Envelope method of randomisation was followed in this prospective in-vivo study. In each subject, one of the first premolar brackets was debonded using a debonding plier and polished following standard protocols. Envelope method of randomisation was used to determine the side of the premolar to be debonded first. Patient was advised to use fluoridated (Group I) dentifrice for the first 15 days, then the first premolar was covered with a heavy-bodied putty cap, extracted and subjected to atomic force microscopy (AFM). Contralateral first premolar was then debonded and polished using similar protocol, and patient was advised to use nano hydroxyapatite dentifrice (Group II) for next 15 days. The premolar was then extracted and analyzed for surface roughness using AFM. The remineralizing potential of dentifrices was assessed by evaluating surface roughness parameters of the two groups and were compared using a two-sample *t* test.

**Results:**

A significant difference was found amongst Group I (Fluoridated dentifrice) and Group II (NanoHAP dentifrice) (*p* > 0.001***) for enamel surface roughness variables which reflect remineralising potential of dentifrices. Group II showed significantly lesser value of surface roughness characteristics.

**Conclusions:**

NanoHAP dentifrice was shown, after 15 days, to be superior to fluoridated dentifrice in remineralising enamel post orthodontic debonding.

## Introduction

Bonding is the fundamental step to a successful orthodontic treatment. Etching being the primary procedure in bonding, creates micro-morphological variation on the resistant enamel surface [1]. At the end of treatment, when orthodontic attachments are debonded, a porous enamel surface with micro-cracks, fractures and retained resin tags remain [2, 3]. Loss of enamel layer ranging up to 52–55 μm due to orthodontic debonding has been reported previously [4, 5]. These micro-damages result in an enamel surface which is rougher and is at further risk for demineralization [2, 3].

Residual resin removal using bur and polishing the enamel surface takes us a step closer to restoring to pre-treatment enamel surface conditions, however it does not fully restore the enamel surface [3, 6–8]. Fluorides [9], compounds of novamin [10], pro-arginine [11], amorphous calcium phosphates [12, 13], hydroxyapatite agents [14, 15] are occasionally prescribed after orthodontic treatment completion for utilising their remineralisation potential.

Fluoridated dentifrices with a fluoride concentration ranging from 1000 to 1450 ppm are commonly used as they are known to decrease demineralization by promoting formation of fluorapatite [16]. However, this acid resistant layer seems to prevent diffusion of remineralising ions into subsurface layers, thus complete remineralization process to depth of lesion does not take place [17]. Apart from professional chair-side application of high concentration fluoride, studies have also assessed potential of high concentration fluoride dentifrice in remineralising carious lesions and reducing the prevalence of white spot lesions post-orthodontic debonding [18–20]. Contrasting results have been displayed regarding the same and recent literature a low remineralizing potential of high fluoride preparations compared to regular fluoride [21]. Increased fluoride content also possesses increased risk of fluorosis. Alternatively, non-fluoridated dentifrices have been suggested as aforementioned.

Hydroxyapatite crystals make up the fundamental and major inorganic fragment of matured enamel. Synthetic forms of hydroxyapatites (HAP) have been used for restoring enamel surfaces. Application of nanotechnology as nanomaterials has surfaced well in the field of orthodontics from nanoparticle coated orthodontic attachments, resin adhesive with nanofillers like silver and nano apatites [22–24]. A more biomimetic form of hydroxyapatite having particle size scaled within nanometers known as nanohydroxyapatite have been successfully used to remineralise enamel surface [25]. Apart from being more biocompatible and bioactive, nano hydroxyapatite crystals have shown to have an appreciable affinity and adaptability towards enamel than a conventional apatites, reason being an increased surface area due to smaller particle size of nano hydroxyapatite crystals. Layers of nanoHAP deposited on the surface of enamel are extremely resistant to acidic solutions and therefore can protect the underlying enamel from future demineralisation [18, 26].

Perusing the enamel surface for surface roughness various methods such as use of stereo microscope, scanning electron microscope (SEM), contact profilometer or AFM have been used [27, 28]. However, the SEM results are not reliable, are subjective and doesn't give a quantitative evaluation. Alternatively, the atomic force microscope produces multiple conspicuous scans and is suitable to assess the nano irregularities on any surface [29].

The loss of enamel surface structure owing to orthodontic procedure is inexorable and sequelae that follow it are also unavoidable. Therefore, it is of clinical importance to evaluate remineralising effects of nano hydroxyapatite dentifrice post-debonding. There is no updated literature that tested and compared the biomimetic effects of nano-hydroxyapatite with that of fluoridated dentifrice after orthodontic debonding. Few in-vitro studies have reported on the role of NanoHAP in reducing surface roughness after orthodontic debonding [14]. However, till date no in-vivo study has been conducted which reaffirms the finding of the in-vitro studies.

The present study was conducted (1) To evaluate the hypothesis that no significant difference exists in the remineralising potential of NanoHAP dentifrice and fluoridated dentifrice after orthodontic debonding, (2) To estimate the enamel parameters following use of nano Hydroxyapatite dentifrice, post orthodontic debonding.

## Methods

Ethical clearance was obtained by the Scientific review Board of Saveetha Institute of Medical and Technical Sciences (IHEC/SDC/ORTHO-1806/20/45). Patients requiring extraction of both maxillary first premolars for the purpose of orthodontic treatment were listed. 30 subjects who were willing to participate in this split mouth trial were enrolled. In total 60 premolars of 30 subjects undergoing orthodontic treatment were contained for the study. Each subject consented to participate in the study by signing a written consent form.

The inclusion was performed on the basis of visual assessment of the compactness of labial surface i.e., no surface micro-fractures/pits, enamel hypoplasia, caries, pigmented surfaces as in smokers, and any restorative procedures involving buccal aspect of bicuspids.

Enamel surfaces were rendered clean and buffed using a rubber-cup topped with pumice paste, in a low-speed. The surface was parched using compressed air (oil-free). The bicuspids were subjected to 37% phosphoric acid gel (D-tech etching gel, D-tech, India) for 10 s, the etchant was washed off completely and the surface was air-dried till a frosty white appearance was seen. Etched enamel surface was painted with a fine coat of primer (OrthoSolo, Ormco) and the metal bracket base (3 M, Unitek Gemini MBT Brackets) was coated with resin adhesive. The brackets were then placed, firmly pressed and excess composite flash was removed. Bonding was accomplished by light- curing bracket for 12 s, each edge cured for 3 s.

After 24 h (maturation period of composite) of bonding, one side of the premolar bracket was debonded using a debonding plier. Mesial and distal wings of the brackets were squeezed together to debond. The premolar to be debonded was determined by the Envelope method of randomisation for each patient. After debonding a 12-fluted tungsten carbide bur (Komet Adhesive Remover, Germany) was used in slow-speed with coolant for removal of remnant resin adhesive from enamel surface. After adhesive resin removal, the surface was polished using pumice slurry coated onto a rubber cup. Under direct illumination of chair-light, meticulous inspection of enamel surface was performed to ensure no resins remain. An experienced orthodontist performed all aforementioned procedures on all subjects and a new polishing inventory was used for every patient including the tungsten carbide bur and polishing cup. Post polishing treatment, fluoridated dentifrice (Amflor dentifrice, Group Pharmaceuticals Ltd, India) was conferred to the participating subject. Subject was given following instructions adhering to the manufacturer’s guidelines:

- Subject was directed to use fluoridated paste regularly for 15 days.
- Paste had to be massaged on the enamel surface for a minimum of 2 min, twice a day for 15 days.
- After application the patient was advised to rinse the paste with water.

**Fig. 1:**
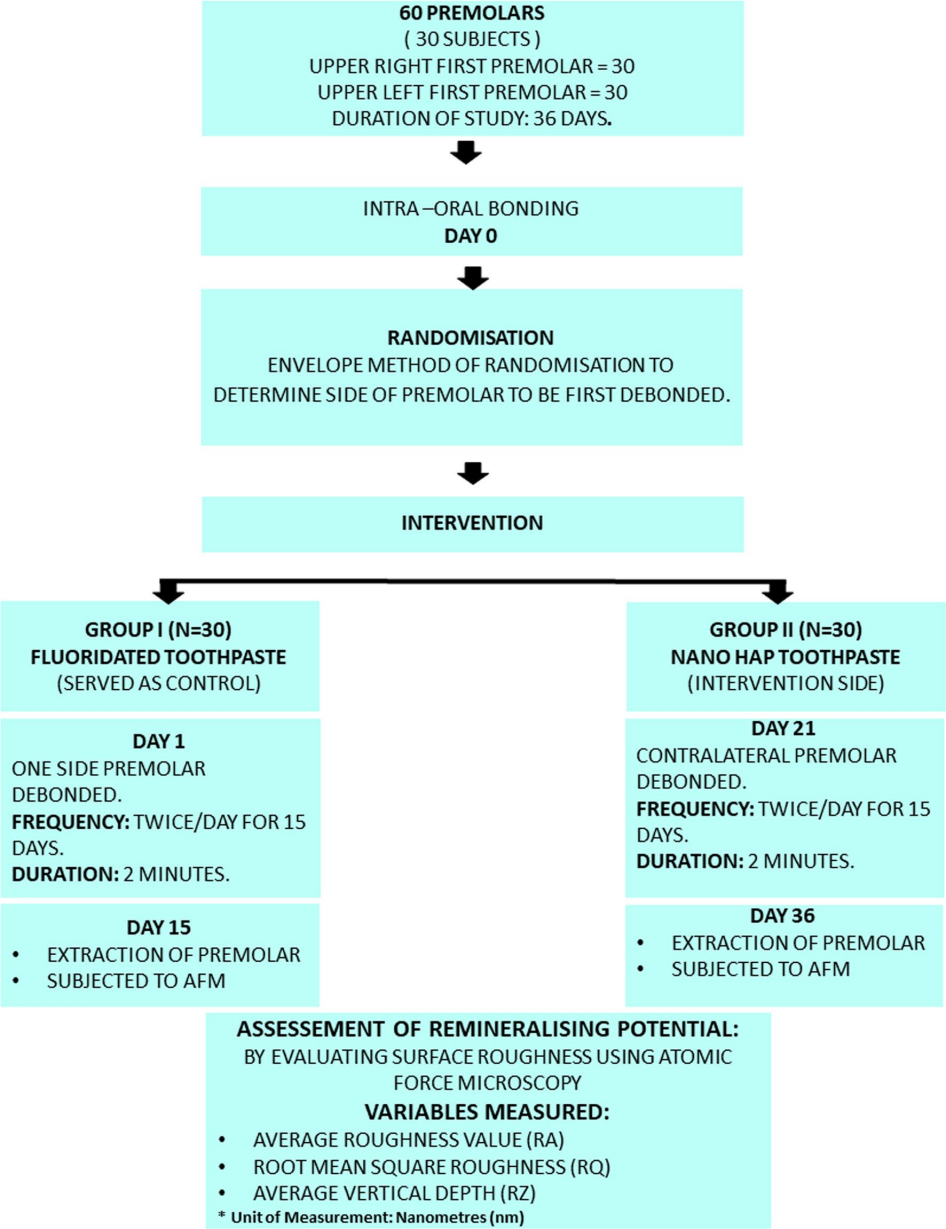
Outflow of the study protocol step-by-step

After 15 days the patient was recalled, the debonded premolar tooth was then subjected to extraction. After a week, the contralateral first premolar was then debonded and polished using the same protocol as aforementioned. NanoHAP dentifrice (Apagard Premio Toothpaste, Japan) was given to the patient and similar protocol for application was followed by the patient for the next 15 days. 15 days later, the patient was recalled and premolar was extracted. The study protocol has been briefly outlined in Fig. 1.

**Fig. 2:**
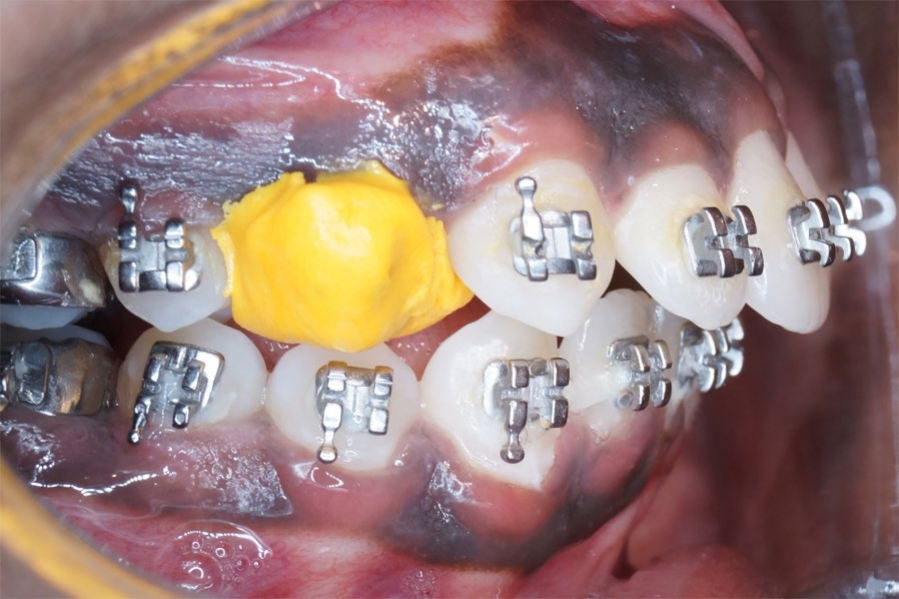
Putty shielded premolar to prevent enamel surface alterations from extraction forceps

In order to circumvent enamel surface damage and surface alterations due to extraction forceps, the premolars were shielded with a heavy-bodied putty impression material before extraction (Fig. 2).

The extraction was done with the putty-shield. After extraction, the root surface was cleaned and distilled water was used for storage of samples till it was transported to the lab where AFM analysis was performed the very same day of extraction. All the premolars were subjected to AFM only once i.e., following respective intervention. A total of 60 premolars received indistinguishable debonding and polishing protocol, correlative pre-extraction preparation and a similar storage and transportation facility.

**Fig. 3:**
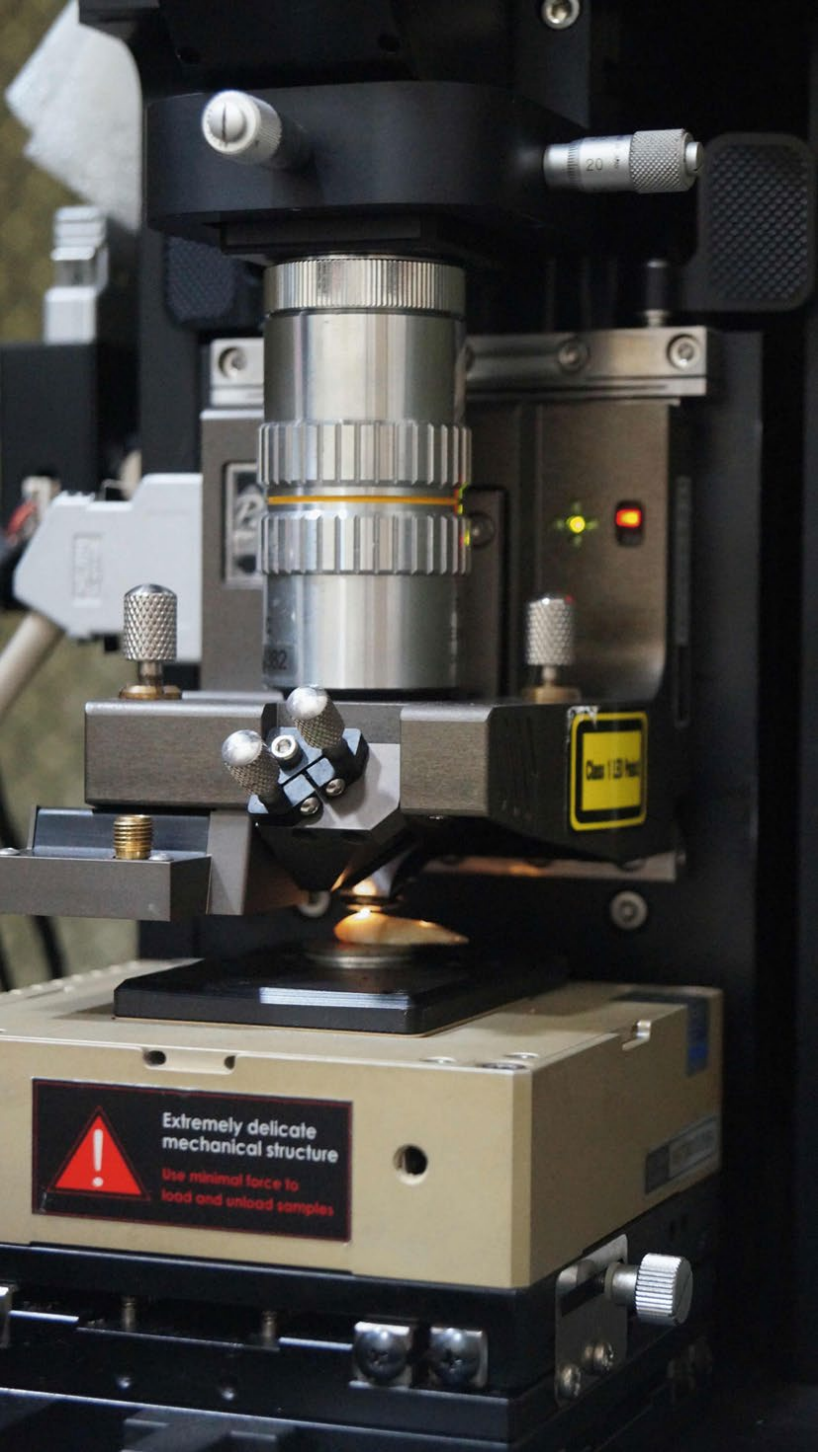
Prepared sample being subjected to atomic force microscopy

**Fig. 4.**
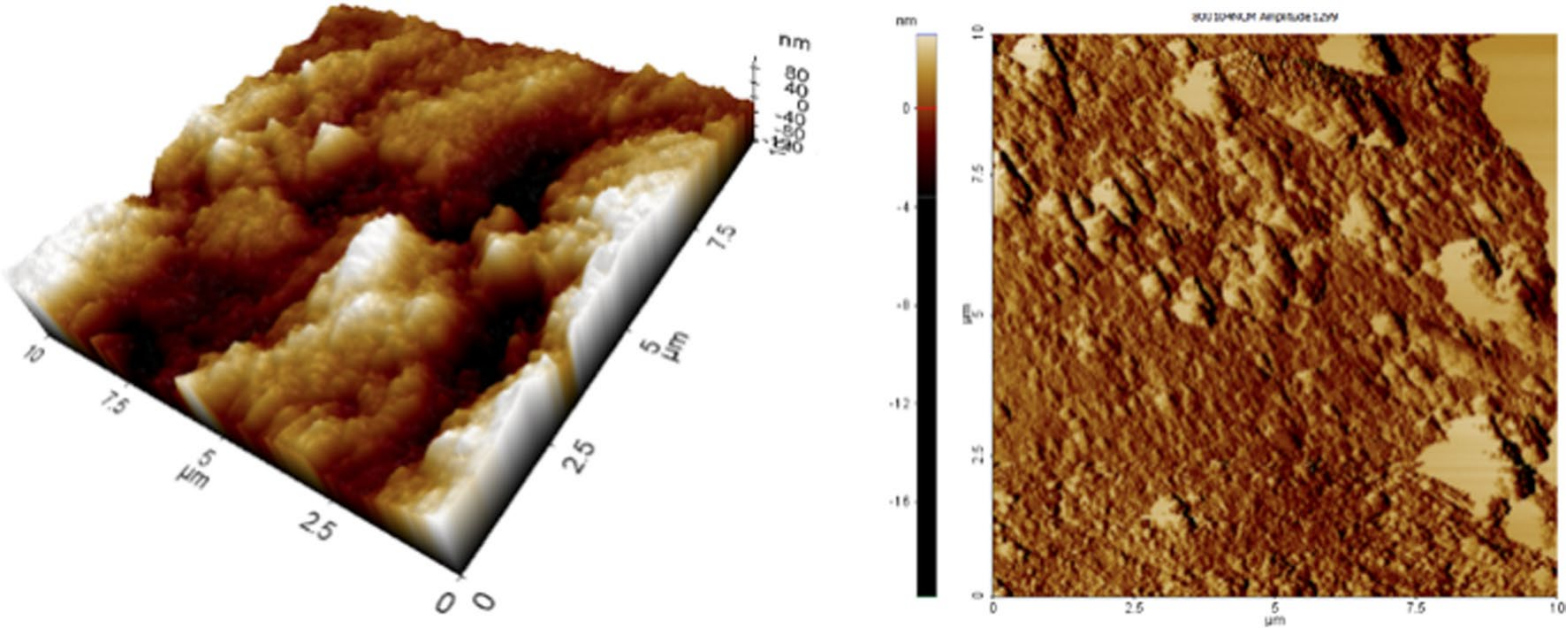
3D and 2D AFM images

The Atomic Force Microscopy (AFM XE-100, Park System, Park Europe, Mannheim, Germany) was executed in contact mode to register the surface topography of enamel (Fig. 3). Atomic Force microscope is incorporated with a scanner which records in the range of 100 μm × 100 μm × 15 μm in *x*, *y*, and *z* axis in order. Scanning was done at 3 points and at each point 2D and 3D scans were obtained at a resolution of 256 × 256 pixels. Each scan measured 10 μm Average of three recorded readings was taken for performing statistical analysis (Fig. 4). Three roughness parameters were recorded in nanometers (nm) as mentioned:

- Average roughness value (Ra): it is the average height of crests and troughs of valleys from the mean line. This signifies gross surface roughness.
- Root mean square roughness (Rq): distribution of heights of crests and troughs in relation to the mean line.
- Rz: it is the vertical length from height peak to lowest valley of five sampling lengths, with the evaluation length.

### Statistics analysis

All the statistics were performed using SPSS software (IBM SPSS Statistics for Windows, Version 26.0, Armonk, NY: IBM Corp. Released 2019). Normality of data distribution was evaluated using Kolmogorov–Smirnov and Shapiro–Wilks test. Parametric tests were used to evaluate data for roughness. Inter-group comparison was performed using a two-sample *t* test. Significance level was fixed at 5% (*α* = 0.05).

## Results

The Normality tests Kolmogorov–Smirnov and Shapiro–Wilks test results reveal that all variables (Ra, Rq, and Rz) follow normal distribution. Independent *t* test revealed that a statistically significant difference was found amongst the two groups for the values of Ra, Rq and Rz. The values were significantly lesser for Group II. (*p* < 0.001) (Table 1).

**Table 1:** Means, standard deviation, and *t* test results of surface roughness measurements (nm = nano meters) for both groups

Variable	Groups	*N*	Mean ± SD (nm)	*p* value
Ra	Group I	30	90.47 ± 6.58	< 0.001***
	Group II	30	28.53 ± 6.13	
Rq	Group I	30	101.16 ± 8.30	< 0.001***
	Group II	30	27.56 ± 6.03	
Rz	Group I	30	716.55 ± 70.16	< 0.001***
	Group II	30	278.25 ± 59.11	

***Statistically significant

Value for Ra was significantly higher for Group I (90.47 ± 6.58 nm) compared to Group II (28.53 ± 6.12 nm). Similarly, mean values were higher for Rq and Rz in Group I being 101.16 nm and 716.55 nm respectively. Whereas, significantly lesser mean values were recorded for Group II. Details of mean, SD and significance have been mentioned in Table 1.

## Discussion

Enamel, being crucial clinically brings along domineering distress in terms of bonding–debonding procedure. Increased enamel surface roughness after debonding serves as a niche for bacterial plaque, accumulation of the pigments, and decalcification, situations that are likely to cause esthetic problems [30]. Unavoidable modifications of enamel surface like loss of enamel, deteriorating surface characteristics, and discoloration post-orthodontic treatments have been reported in previous literature [31, 32].

Several remineralising agents have been used ranging from concentrated fluoride pastes and varnishes, CPP-ACP, pro-argin and nanoHAP serum. A study comparing remineralising effects of fluorides and CPP-ACP demonstrated no significant difference between two exists in terms of remineralising effect [12]. Arginine dentifrice has shown better remineralising potential compared to a fluoridated dentifrice [33]. Novamin has also shown effective remineralization [12] potential compared to a regular fluoridated compound [34]. Studies have also shown that synthetic hydroxyapatite dentifrices have a comparable remineralising potential than conventionally used fluoridated dentifrices [35]. However a biomimetic alternative to HAP, the NanoHAP is chemically and structurally similar to inorganic structure of enamel and has proved to be superior in remineralising the enamel surface than conventional HAP [36]. The nanoHAP particles display elevated surface energy and strong bonding towards the enamel surface [37]. Current study investigated remineralising potential by measuring the surface roughness value following use of NanoHAP dentifrice post-debonding and compared it to a control fluoridated group.

The results of the current study reported a significant difference in enamel surface roughness parameters amongst the two groups following application of both dentifrices for 15 days. As per the manufacturer’s instructions, NanoHAP formulation reaches its greatest effectiveness if applied daily for a minimum of 2–3 min up to a minimum for 10 days/month. A bi-weekly protocol was therefore adopted for this study.

The values for surface roughness were lower significantly in the NanoHAP group in comparison to the fluoridate group. Previous studies by Takikawa et al. [38] and Toko et al. [39] have claimed that NanoHAP restores the enamel surface to its original state. In another study by Scribante et al. [40] they assessed for visual remineralisation, bond strength and hardness of enamel post-orthodontic debonding. In their in-vitro experiment visual effects were recorded using an optical microscope. They concluded that application of the Nano hydroxyapatite solution induced a significant in vitro reduction of demineralised areas. Present study cannot claim so, as the AFM readings were taken only at a one-time point, but the NanoHAP group has shown better surface characteristics than fluoridated dentifrice.

Present study utilized AFM to analyze topographic characteristics of enamel. AFM was considered appropriate to obtain detailed three-dimensional surface characteristics in nanometers. Principal advantage of AFM over SEM is that AFM provides a quantitative information [41].Scanning electron microscopy can also be used to evaluate surface roughness but the SEM photomicrographs are subjective and not reliable as it does not provide quantitative evaluation of surfaces and therefore not suitable for comparative assessment enamel topography [42, 43].An alternative to SEM is AFM as this technology generates high resolution multiple mechanical scans in range of nanoscales. Additionally, AFM requires minimal sample preparation, generates 2D and 3D images simultaneously and allows for re-examination of samples as required [28, 44].

The present study is a novel in-vivo study where an attempt to estimate remineralising potential of NanoHAP dentifrice on surface roughness of enamel was made. Since previous literature has already reported an invariable loss of enamel surface post debonding, it was assumed that the values of baseline surface roughness parameters were high as recording the same was not feasible, study being an in-vivo trial. In-vitro setup being fully controlled does not recreate the natural oral environment, and the results can therefore not always be accurately taken for clinical consideration. The study was designed as a split-mouth trial in order to eliminate an inter-individual variability from the estimates of the treatment effect. However, to avoid any cross over effect the interventions were spaced out in time and only when the application of fluoridated dentifrice was completed after 15 days and the premolar was extracted, the contralateral premolar was debonded and intervention in form of NanoHAP dentifrice was introduced.

The hypothesis of the study was rejected, as a significant difference was found in the remineralising effects of NanoHAP dentifrice and fluoridated dentifrice after orthodontic debonding. Remineralising effects of NanoHAP dentifrice were found to be significantly superior to routine fluoridated dentifrice.

AFM acquires surface topographic images of only a smaller area, which could limit true representation of a wider area involved. To minimise this limiting factor, for each sample a minimum of three points were assessed for recording surface roughness and the average of the three readings were utilised for performing statistics. In future prospects, studies with larger sample size incorporating multiple interventions to assess and grade their remineralization potential can be carried out.

## Conclusions

- There is a significant difference in fluoridated dentifrice and NanoHAP dentifrice in reducing the enamel surface roughness.
- The NanoHAP dentifrice is superior to fluoridated dentifrice in restoring the enamel surface post orthodontic debonding.
- Routine use of NanoHAP dentifrice bi-weekly, twice a day following orthodontic treatment will help reduce post-debonding roughness and sensitivity.

## Data Availability

The datasets used and/or analysed during the current study are available from the corresponding author on reasonable request.

## Abbreviations

NanoHAP: Nano hydroxyapatite
AFM: Atomic force microscope
Nm: nanometer
